# miR-4687-5p Affects Intracellular Survival of *Mycobacterium tuberculosis* through Its Regulation of NRAMP1 Expression in A549 Cells

**DOI:** 10.3390/microorganisms12010227

**Published:** 2024-01-22

**Authors:** Chaoqun Meng, Guangxin Chen, Yue Liu, Da Wen, Jia Cui, Li Dong, Zhiqiang Yang, Hangting Meng, Yuanting Gao, Jiao Feng, Xiaogang Cui, Changxin Wu

**Affiliations:** 1 The Key Laboratory of Medical Molecular Cell Biology of Shanxi Province, Institute of Biomedical Sciences, Shanxi University, 92 Wucheng Road, Taiyuan 030006, China; mcqsxdx@163.com (C.M.); chengx@sxu.edu.cn (G.C.); yueliu@sxu.edu.cn (Y.L.); wdbio@outlook.com (D.W.); cuijia060606@126.com (J.C.); dongli@sxu.edu.cn (L.D.); yzq2021@sxu.edu.cn (Z.Y.); menghangting@163.com (H.M.); sxugyt@163.com (Y.G.); fengjiao2018@sxu.edu.cn (J.F.); 2 The Key Laboratory of Chemical Biology and Molecular Engineering of Ministry of Education of China, Institute of Biotechnology, Shanxi University, Taiyuan 030006, China; 3 The Key Laboratory of the Prevention and Control of Major Infectious Disease of Shanxi Province, Shanxi University, Taiyuan 030006, China

**Keywords:** miR-4687-5p, NRAMP1, *Mycobacterium tuberculosis*, apoptosis, proliferation

## Abstract

Tuberculosis (TB), as one of the leading causes of death, poses a serious predicament to the world. MicroRNAs (miRNAs) play a role in the post-transcriptional regulation of gene expression. It has been reported that the expression of miRNAs changes upon mycobacterial infection; the screening and identification of miRNAs regulating the expression of genes could benefit our understanding of TB pathogenesis and generate effective strategies for its control and prevention. In this study, luciferase assays showed that miR-4687-5p is bound to the 3′-untranslated region of natural resistance-associated macrophage protein 1 (NRAMP1). Additionally, we found a significant increase in miR-4687-5p expression in *Mycobacterium tuberculosis* (*Mtb*)-infected A549 cells. Concomitantly, we detected a reduced level of NRAMP1 expression, suggesting that *NRAMP1* is one of the targets of miR-4687-5p. Infection experiments evidenced that the transfection of miR-4687-5p induced a decrease in NRAMP1 expression and increased intracellular *Mtb* loads post-infection, indicating that miR-4687-5p promotes the intracellular survival of *Mtb* through its downregulation of the NRAMP1 protein level. We also found that the transfection of miR-4687-5p induced increased apoptosis and decreased cell proliferation post-infection with *Mtb*. The results presented in our study suggest that miR-4687-5p may be indicative of the susceptibility of *Mtb* infection to humans and could act as a potential therapeutic target for tuberculosis treatment.

## 1. Introduction

Tuberculosis (TB) is one of the most fatal infectious diseases, a major threat to human health, and a significant cause of death worldwide. TB is caused by *Mycobacterium tuberculosis* (*Mtb*), which spreads when infected people expel bacteria into the air (through coughing). Approximately a quarter of the global population is estimated to be infected with TB [1]. The bacterium has unique resistance to many antimicrobial agents and is a major global health problem due to the emergence of multidrug-resistant strains, which cause drug-resistant TB (DR-TB). Therefore, in order to fully implement the World Health Organization’s End TB Strategy, new drugs or effective strategies with which to treat drug-resistant strains and shorten the treatment duration are crucial for patients with TB, especially those with DR-TB.

MicroRNAs (miRNAs) are small, non-coding, single-stranded RNAs that are approximately 22 nucleotides long, and they play a crucial role in regulating gene expression at the post-transcriptional level [2,3].MiRNAs also precisely regulate target genes in immune cells by modulating the expression of proteins at the post-transcriptional level [4,5]. Additionally, miRNAs participate in cell proliferation, apoptosis, the regulation of immune response, and physiological and biological processes [6]. In recent decades, it has been reported that miRNAs significantly manipulate the innate immune response against TB through their regulation of multiple signaling pathways during the interactions of *Mtb* and the host, while miRNAs contribute to *Mtb* virulence and the host’s immune evasion [7,8,9,10]. The screening and identification of miRNAs that strongly regulate a specific gene that significantly involves host immune responses to *Mtb* infection or human susceptibility to TB has attracted interest in research, as it may lead to more potential miRNAs as targets for the development of an effective therapeutic strategy for tuberculosis in humans through different mechanisms.

Natural resistance-associated macrophage protein 1 (NRAMP1), also named solute carrier family 11A member 1 (SLC11A1), is expressed in macrophages, lymphocytes, and lung tissues in humans [11]. In murine, *Nramp1* knockout causes a high susceptibility to infection with several intracellular bacteria, including *Salmonella, Leishmania*, and *Mycobacterium* [12]. NRAMP1 is highly conserved, containing 12 transmembrane domains [13,14]. NRAMP1 limits the growth and survival of *Mtb*. It has been reported that single nucleotide polymorphisms (SNPs) of SLC11A1 at 5′(GT)nINT4, D543, and 3′-untranslated region (UTR) are strongly associated with TB susceptibility in humans [15]. Furthermore, SNPs in SLC11A1 have also been associated with a variation in gene expression; the expression of SLC11A1 with some particular SNPs in TB patients could influence the function of other related genes and impair immune responses to *Mtb* infection [16]. It is expected that the change in or creation of particular SNPs in miRNA targeting sequences may impact the host’s susceptibility to TB, or the manipulation of miRNA expression may significantly change human susceptibility to TB.

It has been reported that miR-4687-5p acts as a potential diagnostic biomarker of amyotrophic lateral sclerosis [17]. However, the function and mechanism of TB is still unknown. In this study, we aimed to analyze the expression of miR-4687-5p in the *Mtb*-infected cell to investigate whether miR-4687-5p influences susceptibility to TB in humans via targeting NRAMP1.

## 2. Materials and Methods

### 2.1. Cell and Bacteria Culture

A549, a human alveolar epithelial cell line, was obtained from BeNa Co (Beijing, China) and maintained in a 1640 medium (Boster, Wuhan, China) containing 10% fetal bovine serum (FBS) (Gibco, Grand Island, NE, USA). The HEK293T cell line was purchased from BeNa Co (Beijing, China) and cultured in a DMEM medium (Boster, Wuhan, China) supplemented with 10% heat-inactivated FBS (Gibco, Grand Island, NE, USA). All cells were cultured in a humidified incubator with CO_2_ (5%) at 37 °C.

H37Ra, an avirulent *Mtb* strain, was a gift from Professor Yujiong Wang (School of Life Sciences, Ningxia University). The *Mtb*-expressing red fluorescent protein, pTEC27, was provided by Lalita Ramakrishnan (Department of Medicine, University of Cambridge, UK). *Mtb* was maintained at 37 °C in a Middlebrook 7H9 medium (BD, New York, NY, USA) with 10% acid albumin dextrose catalase (ADC) (BD, New York, NY, USA).

### 2.2. Cell Transfection

A549 cells were inoculated in 12-well cell culture plates and transfected with a miR-4687-5p mimic negative control, miR-4687-5p mimic, miR-4687-5p inhibitor negative control, and miR-4687-5p inhibitor. We used the Lipofectamine 3000 Transfection Kit (Invitrogen, Carlsbad, CA, USA) according to the procedures outlined in the instructions. The synthetic miR-4687-5p mimic (5′-CAGCCCUCCUCCCGCACCCAAA-3′) and miR-4687-5p inhibitor (5′-UUUGGGUGCGGGAGGAGGGCUG-3′), miR-4687-5p mimic negative control (5′-UUCUCCGAACGUGUCACGUTT-3′), and miR-4687-5p inhibitor negative control (5′-CAGUACUUUUGUGUAGUACAA-3′) were provided by GenePharma (Shanghai, China).

### 2.3. Luciferase Reporter Assay

MiRNA, which regulates NRAMP1, was predicted using several databases. HEK293 cells were inoculated in 24-well cell culture plates and co-transfected with the luciferase reporter plasmid (Sangon Biotechnology, Shanghai, China) of wild-type (NRAMP1-wt), and mutant NRAMP1 in the 3′-UTR (NRAMP1-Mut), or an empty vector plasmid (50 ng/well) along with the miR-4687-5p mimic, miR-4687-5p inhibitor, or using a negative control with the Lipofectamine 3000 reagent. After transfection for 48 h, cells were used to analyze luciferase activity using a TransDetect^®^ Double-Luciferase Reporter Assay Kit (TransGen Biotech, Beijing, China) via a Dual-Light Chemiluminescent Reporter GeneAssay System (BioTek, Winooski, VT, USA).

### 2.4. Cell Proliferation Assay

A549 cells were inoculated in 12-well cell culture plates and transfected with the miR-4687-5p mimic, miR-4687-5p inhibitor, scrambled control for 48 h, and then incubated with a 2× 5-ethynyl-2′-deoxyuridine (EdU) solution (20 μM) using the EdU Kit (Beyotime, Shanghai, China) following the procedures outlined in the instructions. After incubating for 2 h, the medium was removed, and 1 mL of 4% paraformaldehyde (PFA) was added to the cells for 15 min, and then washed 3 times with 3% bovine serum albumin (BSA), Triton X-100 (0.3%) was added to each well and incubated for 15 min at room temperature. Then, the cells were washed 3 times with 3% BSA and incubated with 0.5 mL of click solution for 30 min at room temperature, protected from light. Each well was washed 3 times with 3% BSA. A549 cells were stained with 1× Hoechst 33342 for 10 min with protection against light damage at room temperature. We quantified the results using a Nikon TE300 fluorescent microscope (Nikon Corporation, Tokyo, Japan).

### 2.5. Cell Apoptosis Test

A549 cells were inoculated in 6-well cell culture plates and transfected 48 h with a miR-4687-5p mimic, miR-4687-5p inhibitor, and scrambled control. The cells were stained with Annexin V(AV)-AbFluor™ 488/propidium iodide (PI) (Abbkine, Beijing, China) following procedures outlined in the instructions. Early and late phases of apoptosis were measured using flow cytometry.

### 2.6. Mtb Infection Assay and CFU

A549 cells were inoculated in 12-well cell culture plates at the desired density and were transiently transfected with the miR-4687-5p mimic, miR-4687-5p inhibitor, and scrambled control. After transfection for 48 h, the cells were infected at a multiplicity of infections (MOIs) 1:10 with *Mtb* for 24 h. The cells were then washed with PBS to remove extracellular bacteria. PBS containing 0.1% Triton X-100 was added to lyse cells for 10 min; the lysis solution was cultured on Middlebrook 7H10 agar plates with 10% oleic acid albumin dextrose catalase (OADC) enrichment (BD, New York, NY, USA). The plates were incubated for about 3 weeks at 37 °C. We tested the viability of intracellular bacteria by enumerating colony-forming units (CFUs).

### 2.7. Western Blotting

Cells were lysed using a radio immunoprecipitation assay (RIPA) buffer (Boster, Wuhan, China) containing a protease inhibitor. The total protein was quantified using the Pierce BCA Protein Assay Kit (Thermo Fisher, Waltham, MA, USA) following the procedure outlined in the instructions. Proteins were separated using 12.5% polyacrylamide gel and transferred to 0.45 μm of polyvinylidene fluoride (PVDF) membranes using a Trans-blot Turbo device (Bio-Rad, Hercules, CA, USA) at 75 V on ice for 1.5 h. The membranes were blocked with 5% skimmed milk (Solarbio, Beijing, China) for 1 h at room temperature. Then, the primary antibody was used to incubate the membranes overnight at 4 °C. The primary antibody and dilution rate are described as follows: anti-NRAMP1 (1:500) (Novus, Wuhan, China), β-actin (1:20,000) (Proteintech, 66009-1-Ig, Rosemont, IL), caspase-3 (1:1000) (Abclonal, Wuhan, China), proliferating cell nuclear antigene (PCNA) (1:5000) (Elabscience Biotechnology Co. Ltd., Wuhan, China). Next, the membranes were incubated with the secondary antibody horse radish peroxidase (HRP) goat anti-rabbit IgG (1:10,000) and HRP goat anti-mouse IgG (1:10,000) (ABclonal, Wuhan, China) at room temperature for 1 h. Finally, the membranes were scanned using chemiluminescence imaging software (AI600 Control. RDP).

### 2.8. qRT-PCR

RNAiso Plus (TaKaRa,9109, Kyoto, Japan) was used to extract the total RNA from A549 cells following the procedures outlined in the instructions. Nanodrop (Thermo Fisher, Waltham, MA, USA) was used to quantify the concentration of RNA, and 1000 ng of RNA was used to generate cDNA using the PrimeScript^TM^ RT Master Mix (TaKaRa, RR036A, Kyoto, Japan). Quantitative reverse transcription-PCR (qRT-PCR) was performed using triplicates of each sample. The primers were designed and synthesized via Sangon Biotechnology Co. (Shanghai, China). Two X M5 HiPer Realtime PCR Super Mixes (Mei5 Biotechnology, Beijing, China) were used to analyze the levels of NRAMP1 mRNA using the Light Cycler 480 system (Roche, Basel, Switzerland). The primer sequences are described as follows: NRAMP1, forward (F) 5′-CCGCCGAGCAGACATCAGAG-3′ and reverse(R) 5′-GTTGAACGCAGCCTGGTTGG-3′; β-actin, forward (F) 5′-GATGGAAAGTGACCCGCA-3′ and reverse (R) 5′-GAGGAAGACGCAGAGGTTTG-3′.

For miRNA expression analysis, the qRT-PCR of miR-4687-5p was performed using Hairpin-it^TM^ microRNA and a U6 snRNA Normalization RT-PCR Quantitation Kit with GenePharma (Shanghai, China) following the procedures outlined in the instructions. The relative miR-4687-5p levels were normalized to U6 RNA and analyzed using the 2^−ΔΔCt^ method.

### 2.9. Immunofluorescence

A549 cells on slides in 24-well cell culture plates were transfected with the miR-4687-5p mimic, miR-4687-5p inhibitor, and scrambled control for 48 h, and were then infected (MOI) 1:10 with *Mtb* for 24 h. The coverslips mounted with cells were washed 3 times for 5 min each. Next, the cells were fixed with 4% PFA for 15 min at room temperature. DAPI (Sigma-Aldrich, St. Louis, MO, USA) was used to stain the nucleus. Finally, the cells were washed 3 times with PBS. The slides were sealed with *Permount* visualized, and images were acquired using a confocal microscope (Carl Zeiss Meditec AG, Jena, Germany). *Mtb* was observed in red as it was labeled with the expression of tdTomato, and nuclei were shown in blue.

### 2.10. Statistical Analysis

All data are expressed as three independent biological replicates. The experimental results are presented as the mean ± standard error of mean (SEM). Statistical analysis was performed using GraphPad Prism 8.00. Group comparisons were conducted using one-way analysis of variance (ANOVA) followed by the least significant difference test. (* *p* < 0.05, ** *p* < 0.01,*** *p* < 0.001) Multiple comparisons between the groups were performed using Tukey’s post hoc test.

## 3. Results

### 3.1. Identification of NRAMP1 as a Direct Target of miR-4687-5p

**Figure 1:**
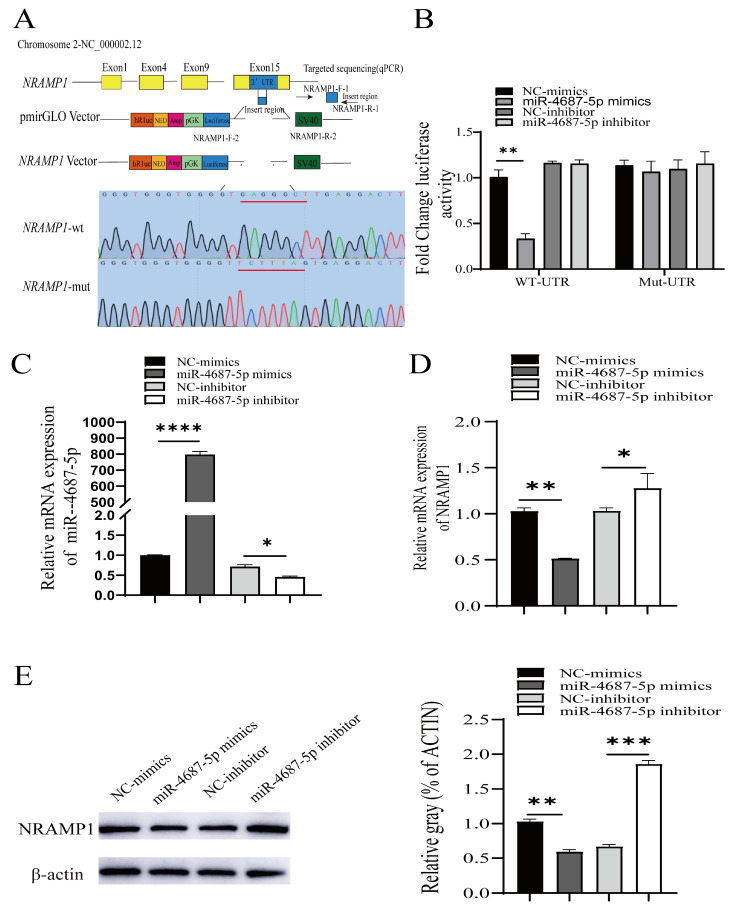
The identification of NRAMP1 as a direct target of miR-4687-5p. (**A**) The pmirGLO vectors contain the predicted 3′UTR target sequences or the mutated reporter sequences (underlined red nucleotides). (**B**) The change in luciferase activity in HEK293T cells. (**C**) qRT-PCR analysis of the expression of miR-4687-5p in transfected A549 cells. (**D**) qRT-PCR measure of the NRAMP1 mRNA levels in transfected A549 cells. (**E**) Western blots to analyze the NRAMP1 protein expression in transfected A549 cells. Experiments were performed at least 3 times, and data are presented as the means ± SD. (* *p* < 0.05, ** *p* < 0.01, *** *p* < 0.001, **** *p* < 0.0001).

To investigate whether miR-4687-5p directly regulates NRAMP1, we cloned NRAMP1 mutation constructs into the pmirGLO vector (Figure 1A). For further validation, a luciferase reporter assay was used to determine whether miR-4687-5p directly targets the 3ʹ-UTR of NRAMP1 mRNA. Constructs harboring the wild-type (wt-UTR) or the mutant NRAMP1 3′ UTR (mut-UTR) were co-transfected with miR-4687-5p mimics, the miR-4687-5p inhibitor, or the corresponding scramble controls into HEK293T cells. Compared to the scrambled control and 3′-UTR, which reported co-treated cells, the 3ʹ-UTR luciferase reporter and miR-4687-5p mimic significantly co-transfected and decreased luciferase reporter activity, whereas no difference was found when the 3ʹ-UTR of NRAMP1 was mutated (Figure 1B). We found that transfection with the miR-4687-5p mimic remarkably promoted the miR-4687-5p mRNA level, while transfection with the miR-4687-5p inhibitor inhibited the miR-4687-5p mRNA level (Figure 1C). To explore the effects of miR-4687-5p on post-transcriptional NRAMP1, we detected the mRNA and protein levels of NRAMP1 in transfected A549 cells. Transfection with the miR-4687-5p mimic dramatically decreased the mRNA and protein levels of NRAMP1 compared to the control group. Conversely, in comparison with the inhibitor-negative control (NC) group, transfection with the miR-4687-5p inhibitor dramatically increased the mRNA and protein levels of NRAMP1 (Figure 1D,E). Taken together, these results demonstrate that miR-4687-5p affects NRAMP1 expression by directly binding to the 3′ UTR region of NRAMP1.

### 3.2. The Role of miR-4687-5p/NRAMP1 on Cell Proliferation and Apoptosis

**Figure 2:**
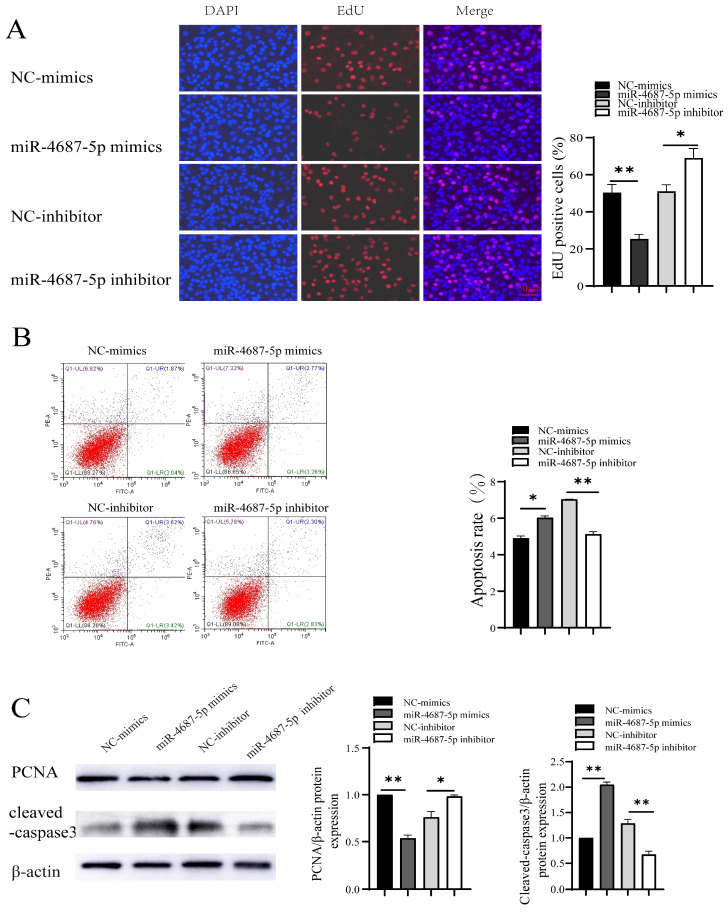
The role of miR-4687-5p and NRAMP1 on cell proliferation and apoptosis. A549 cells were transfected with miR-4687-5p mimics, the miR-4687-5p inhibitor, or corresponding scramble controls. (**A**) EdU assays in transfected A549 cells. Scale bar: 50 μm. (**B**) Flow cytometry analysis of cell apoptosis in transfected A549 cells. (**C**) Western blot and band quantification analysis to determine the PCNA and cleaved-caspase-3 protein level in transfected A549 cells. (* *p* < 0.05, ** *p* < 0.01).

Next, we investigated the effects of miR-4687-5p/NRAMP1 on cell proliferation and apoptosis. The results showed that the overexpression of miR-4687-5p suppressed cell proliferation, whereas the knockdown of miR-4687-5p promoted cell proliferation (Figure 2A). In addition, apoptosis was significantly increased in the miR-4687-5p overexpression of A549 cells, while apoptosis was depressed in miR-4687-5p-silenced A549 cells (Figure 2B). Proliferating the cell nuclear antigen (PCNA) is a cell cycle-associated protein. Similarly, the overexpression of miR-4687-5p decreased the PCNA protein level and increased the cleaved-caspase-3 (a key enzyme that causes cell apoptosis) protein level; the knockdown of miR-4687-5p exerted the opposite effects (Figure 2C). These results highlight the modulatory functions of miR-4687-5p/NRAMP1 on cell growth and apoptosis.

### 3.3. miR-4687-5p Affects Mtb Susceptibility via Targeting NRAMP1

**Figure 3:**
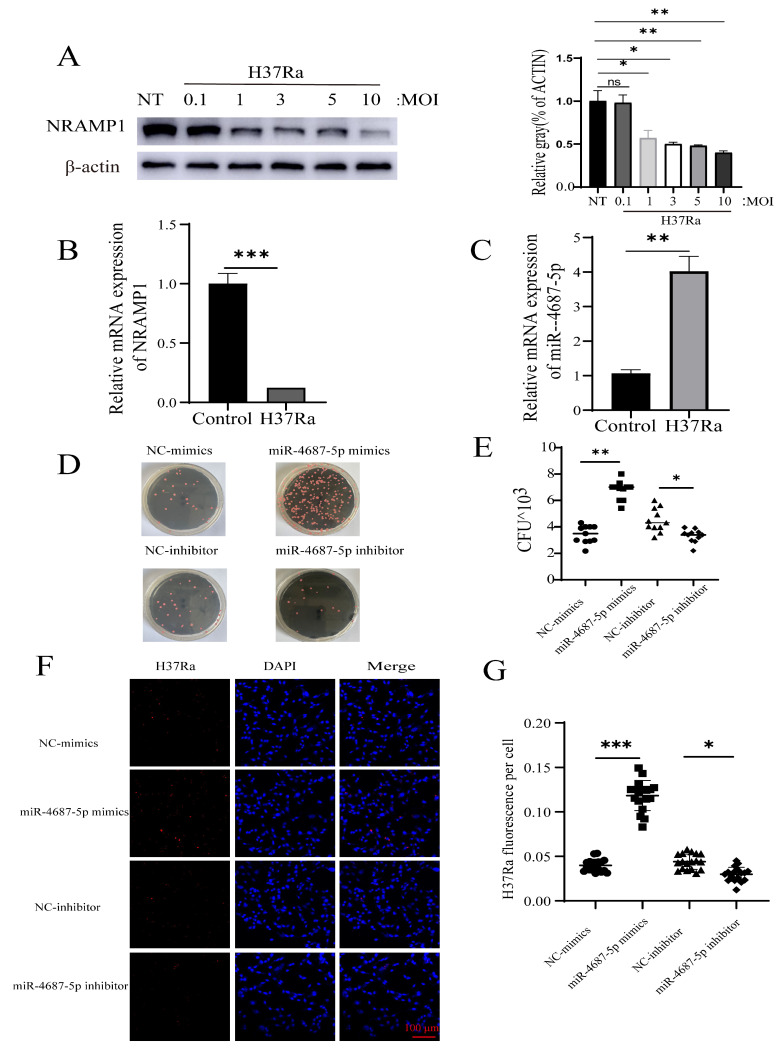
miR-4687-5p affects *Mtb* susceptibility via targeting NRAMP1. (**A**) A549 cells were infected with *Mtb* (with an MOI of 0.1, 1, 3, 5, or 10) for 24 h. NRAMP1 protein levels were determined by Western blot and band quantification analysis. (**B**) qRT-PCR analysis of miR-4687-5p expression in transfected *Mtb* A549 cells or non-infected cells (**C**) qRT-PCR analysis of NRAMP1 mRNA levels in transfected *Mtb* A549 cells or non-infected cells. A549 cells were transfected with miR-4687-5p mimics, the miR-4687-5p inhibitor, or corresponding scramble controls for 48 h, then infected (MOI) 1:10 with *Mtb* for 24 h. (**D**,**E**) Intracellular *Mtb* survival was determined via the CFU assay. (**F**) Immunofluorescence analysis of H37Ra in the infected cells. (**G**) Image J was used to analyze the immunofluorescence intensity of H37Ra in cells. Scale bar: 100 μm. Experiments were performed 3 times, and data are presented as the means ± SD. (* *p* < 0.05, ** *p* < 0.01, *** *p* < 0.001).

To explore the effect of miR-4687-5p in intracellular *Mtb* survival, serial infection experiments were performed. The results demonstrated that A549 cells infected with different MOIs of *Mtb* resulted in an alteration of NRAMP1 expression levels and decreased NRAMP1 expression by approximately 60% at MOI 10 (Figure 3A). We also found that *Mtb* infection induced enhanced miR-4687-5p expression while decreasing theNRAMP1 mRNA level compared with the control group (Figure 3B,C). The colony-forming units counting assay demonstrated that miR-4687-5p increased intracellular *Mtb* survival, whereas the miR-4687-5p inhibitor decreased intracellular *Mtb* survival (Figure 3D,E). We detected the fluorescence intensity of intracellular tdTomato-H37Ra using an LSM 710 confocal microscope and Image J 1.44p software to analyze the fluorescence intensity of tdTomato-H37Ra. The results demonstrate that miR-4687-5p overexpression caused a clear decrease in bacterial uptake, and miR-4687-5p silencing induced a significant increase in bacterial uptake (Figure 3F,G). The above experiments show how miR-4687-5p affects the intracellular survival of *Mtb* via targeting NRAMP1.

### 3.4. The Role of miR-4687-5p/NRAMP1 on Cell Proliferation and Apoptosis Post Mtb Infection

**Figure 4:**
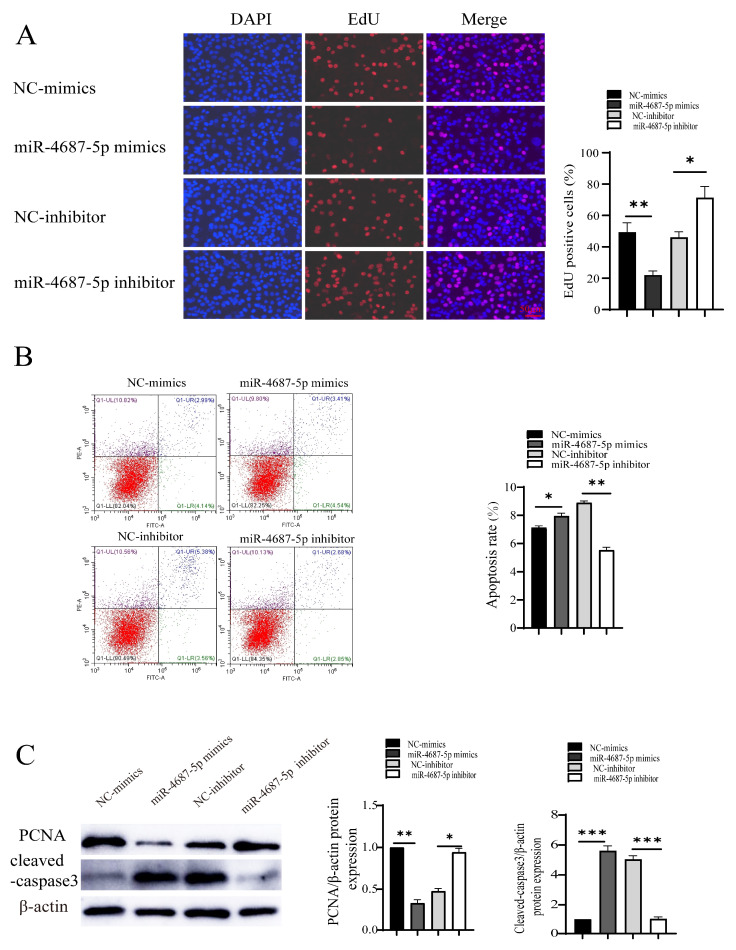
The effect of miR-4687-5p/NRAMP1 on cell proliferation and apoptosis post-*Mtb* infection. A549 cells after transfection with miR-4687-5p mimics, the miR-4687-5p inhibitor, or corresponding scramble controls for 48 h, then infected (MOI) 1:10 with *Mtb* for 24 h. (**A**) EdU assays of transfected A549 cells. Scale bar: 50 μm. (**B**) Flow cytometry analysis of cell apoptosis. (**C**) Western blot and band quantification analysis to determine the PCNA and cleaved-caspase-3 protein level. (* *p* < 0.05, ** *p* < 0.01, *** *p* < 0.001).

We next investigated the effects of miR-4687-5p/NRAMP1 on cell proliferation and apoptosis in *Mtb*-infected A549 cells. The EdU assay results showed that the overexpression of miR-4687-5p suppressed cell proliferation, while the knockdown of miR-4687-5p exerted the opposite effects (Figure 4A). In addition, a high apoptosis level was assayed in miR-4687-5p overexpression cells, while apoptosis was depressed in miR-4687-5p-silenced cells (Figure 4B). Similarly, the overexpression of miR-4687-5p decreased the PCNA protein level and increased the cleaved-caspase-3 protein level, while the knockdown of miR-4687-5p exerted the opposite effects (Figure 4C). These results highlight the modulatory functions of miR-4687-5p/NRAMP1 on cell proliferation and apoptosis post-*Mtb* infection.

## 4. Discussion

TB remains a public health threat, especially in developing countries [15,18]. It is well-known that some host genes play a crucial role in determining human susceptibility and resistance to TB [19,20]. Although macrophages and dendritic cells are primarily colonized by *Mtb*, increasing evidence has revealed that non-phagocytic cells, specifically pulmonary epithelial cells, play a crucial role as a niche for *Mtb* replication and dissemination from the lung to other organs [21,22,23]. Previous studies demonstrated the impact of *Mtb* infection on IL10 and XBP1 in epithelial cells [24,25]. Here, we identified the miR-4687-5p as a determinant influencing intracellular survival of *Mtb* in A549 cells, and investigated the underlying mechanisms. *Mtb* infection can change host epigenetic patterns, the processes of which can change gene expression, but it does not change the DNA sequence, miRNA, etc. [26].

A creative bio-diagnostic miRNA was introduced and has been widely applied in the diagnosis of conditions such as heart disease, psoriasis, cancer, pregnancy, diabetes, and infectious disorders [27]. Moreover, it has been reported that miRNAs can be used as biomarkers for TB diagnosis. Ebert et al. found that some miRNAs can regulate cell differentiation and functions [28]. Moreover, increasing evidence has revealed that miRNAs play crucial roles in regulating innate and acquired immune responses [28,29,30,31]. Lucinda et al. confirmed that the infection of macrophages with *Mtb*-induced miRNA signature changes, including high levels of miR-146a, miR-155, miR-27a, miR-27b, and miR-145 and these miRNAs potentially target immune response-related genes [32]. Some miRNA gene sequences might be crucial for induction resistance or susceptibility to TB [30]. In the current study, we predicted that miR-4687-5p is the upstream miRNA of NRAMP1 using bioinformatics software. We substantiated that miR-4687-5p binds to the 3ʹ-UTR region of NRAMP1 via the luciferase reporter assay (Figure 1). In a previous study, miR-4687-5p was used as a potential diagnostic biomarker, which was upregulated when comparing its metastasis with the N-metastasis group in human non-small cell lung cancer [33]. In this study, we investigated the effect of miR-4687-5p/NRAMP1 on cell proliferation and apoptosis. We found that the increase of miR-4687-5p by transfect mimic inhibited A549 cell proliferation and promoted cell apoptosis by regulating NRAMP1 expression, while silence of miR-4687-5p using its inhibitor showed the opposite effects (Figure 2).

NRAMP1 plays a crucial role in mycobacterial infection in early immune responses [34]. NRAMP1 is associated with diverse infectious diseases and inflammatory diseases [35]. The function of the NRAMP1 protein has not yet been elucidated in humans; however, studies show that it plays a critical role in intercellular microorganisms, where it may modify the phagocytic environment and affect microbial replication [36]. However, an investigation into the mechanisms underlying the manipulation of human susceptibility to TB via *NRAMP1* has not been reported so far.

Understanding the mechanism underlying the effect of miRNA miR-4687-5p on *Mtb* infection and TB development could provide a scientific foundation for developing effective strategies that can prevent and control TB. We found that miR-4687-5p expression was associated with *Mtb* infection and also correlated with NRAMP1 expression; our experiments also clarified that *Mtb* infection induces a reduction in NRAMP1 expression by increasing miR-4687-5p, which downregulates NRAMP1 expression through post-transcription modification machinery. Our previous study showed that the expression level of NRAMP1 affects *Mtb* infection and intracellular *Mtb* survival; here, we demonstrated that miR-4687-5p expression regulates NRAMP1 expression, and NRAMP1 expression affects *Mtb* infection (Figure 3). Autophagic, necrosis, and apoptosis are common forms of cell death processes that play crucial roles in the maintenance and development of balance in tissues [37,38]. In *Mtb*-infected cells, cell apoptosis due to *Mtb* infection is an innate defense mechanism, providing a niche for *Mtb* replication before it escapes into the extracellular milieu [39]. Apoptosis, on the other hand, results in programmed cell suicide, thus limiting the replication of *Mtb* [40]. It has been reported that the regulation of apoptosis affects mycobacterial cell-to-cell spread in vitro, suggesting a clear relationship between *Mtb* infection and apoptosis [38]. An apoptotic marker, active-caspase-3, was found in infected cells [41,42,43]. Therefore, we investigated if miR-4687-5p expression affects proliferation and apoptosis post-*Mtb* infection. Here, we found that a high level of miR-4687-5p expression increased apoptosis; however, it also inhibited proliferation (Figure 4), suggesting that miR-4687-5p is functional as an apoptosis biomarker of *Mtb*-infected cells.

**Figure 5:**
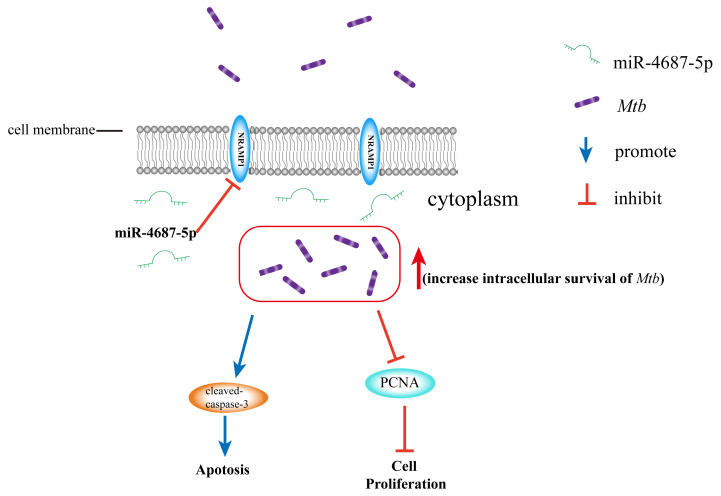
miR-4687-5p downregulates the NRAMP1 expression, resulting in increased intracellular survival of *Mtb*. Additionally, it promotes apoptosis by upregulating cleaved-caspase-3 expression and inhibits cell proliferation by decreasing PCNA expression.

In general, our study found that A549 cells infected with *Mtb* increased the levels of miR-4687-5p and decreased the expression of NRAMP1, subsequently reducing cell proliferation and promoting apoptosis (Figure 5). Our results suggest a link between miRNA expression and *Mtb* infection and demonstrate the molecular mechanism underlying the correlated relationship between miR-4687-5p, NRAMP1 expression, apoptosis, and the intracellular survival of *Mtb* post-infection. These results and information enrich our understanding of TB pathogenesis and should be helpful for the development of drugs, vaccines, and therapies for curing TB.

## 5. Conclusions

In conclusion, miR-4687-5p expression affects the intracellular survival of *Mtb* via miR-4687-5p/NRAMP1/apoptosis; this is realized through high levels of miR-4687-5p expression, low levels of NRAMP1 expression, high apoptosis activity, and more intracellular *Mtb* post-infection. This study suggests that miR-4687-5p may be act as a potential diagnostic or therapeutic biomarker for the effective prevention and control of TB.

## Data Availability

All relevant data are available within the manuscript.

## Abbreviations

ADC	acid albumin dextrose catalase
ANOVA	analysis of variance
BSA	bovine serum albumin
CFU	colony forming units
DR-TB	drug-resistant TB
EdU	5-ethynyl-2′-deoxyuridine
FBS	fetal bovine serum
HRP	horse radish peroxidase
*Mtb*	*Mycobacterium tuberculosis*
MOI	multiplicity of infection
NC	negative control
NT	non-treated
NRAMP1	natural resistance-associated macrophage protein 1
PCNA	proliferating cell nuclear antigen
PFA	paraformaldehyde
PVDF	polyvinylidene fluoride
qRT-PCR	quantitative reverse transcription-PCR
RIPA	radio immunoprecipitation assay
SEM	standard error of mean
SLC11	solute carrier family 11
SNP	the single nucleotide polymorphism
TB	tuberculosis
UTR	untranslated region
